# Upregulation of DUSP6 impairs infectious bronchitis virus replication by negatively regulating ERK pathway and promoting apoptosis

**DOI:** 10.1186/s13567-020-00866-x

**Published:** 2021-01-11

**Authors:** Huan Wang, Dingxiang Liu, Yingjie Sun, Chunchun Meng, Lei Tan, Cuiping Song, Xusheng Qiu, Weiwei Liu, Chan Ding, Liao Ying

**Affiliations:** 1 Waterfowl Viral Infectious Diseases Team, Shanghai Veterinary Research Institute, Chinese Academy of Agricultural Sciences, Shanghai, 200241 P. R. China; 2 Jiangsu Co-Innovation Center for Prevention and Control of Important Animal Infectious Diseases and Zoonoses, Yangzhou, 225009 P. R. China; 3 Guangdong Province Key Laboratory of Microbial Signals and Disease Control and Integrative Microbiology Research Centre, South China Agricultural University, Guangzhou, 510642 P. R. China

**Keywords:** IBV, ERK1/2, DUSP6, virus replication

## Abstract

Elucidating virus-cell interactions is fundamental to understanding viral replication and identifying targets for therapeutic control of viral infection. The extracellular signal-regulated kinase (ERK) pathway has been shown to regulate pathogenesis during many viral infections, but its role during coronavirus infection is undetermined. Infectious bronchitis virus is the representative strain of *Gammacoronavirus*, which causes acute and highly contagious diseases in the poultry farm. In this study, we investigated the role of ERK1/2 signaling pathway in IBV infection. We found that IBV infection activated ERK1/2 signaling and the up-regulation of phosphatase DUSP6 formed a negative regulation loop. Pharmacological inhibition of MEK1/2-ERK1/2 signaling suppressed the expression of DUSP6, promoted cell death, and restricted virus replication. In contrast, suppression of DUSP6 by chemical inhibitor or siRNA increased the phosphorylation of ERK1/2, protected cells from apoptosis, and facilitated IBV replication. Overexpression of DUSP6 decreased the level of phospho-ERK1/2, promoted apoptosis, while dominant negative mutant DUSP6-DN lost the regulation function on ERK1/2 signaling and apoptosis. In conclusion, these data suggest that MEK-ERK1/2 signaling pathway facilitates IBV infection, probably by promoting cell survival; meanwhile, induction of DUSP6 forms a negative regulation loop to restrict ERK1/2 signaling, correlated with increased apoptosis and reduced viral load. Consequently, components of the ERK pathway, such as MEK1/2 and DUSP6, represent excellent targets for the development of antiviral drugs.

## Introduction

Infectious bronchitis virus (IBV) belongs to *gammacoronavirus, coronavirade*, *Nidovirale.* This etiological agent infects domestic fowl and causes a highly contagious respiratory disease with a huge economic impact in the poultry industry [1]. Various IBV strains have been reported worldwide [2], with pathologies ranging from mild respiratory symptoms to severe kidney and oviduct disease [3]. IBV harbors a single-stranded positive RNA genome with a length of ~ 27.6 kb, which encodes polyprotein 1a and 1ab, spike protein (S), 3a, 3b, envelope protein (E), membrane protein (M), 5a, 5b, and nucleocapsid protein (N). Two-thirds of the viral genome encode polyproteins 1a and 1ab, which are proteolytically processed into 15 non-structural proteins (nsp2–16), which are mainly involved in virus replication by forming a replication/transcription complex (RTC). S protein forms trimer on the virus envelope, and is responsible for entry into cells by receptor binding and membrane fusion [4]. M protein and E protein are also on the virus envelope and are involved in virus assembly and budding [5, 6]. E protein is a viroporin which forms an ion channel on the cell membrane and contributes to inflammasome activation and pathogenesis [7–10]. N protein binds to and protects genomic RNA, buried under the virus envelope [11]. 3a, 3b, 5a, and 5b belong to accessory proteins, which probably contribute to virus virulence, host protein translation shut-off [12–16].

Virus replication relies on many functional components in the host cells. Mitogen-activated protein kinase (MAPK) is involved in various cellular activities, such as gene expression, mitosis, cell differentiation, proliferation, and death [17]. The most intensely studied MAPK are extracellular signal-regulated protein kinases 1 and 2 (ERK1/2), p38 kinase, and c-Jun N-terminal kinase (JNK). Among the MAPK signaling pathways, regulation of p38 by coronavirus has been wildly reported, where it plays a critical role during virus infection, including those with mouse hepatitis virus (MHV) [18–20], severe acute respiratory syndrome coronavirus (SARS-CoV) [21–25], feline coronavirus (FCoV) [26], Infectious bronchitis virus (IBV) [27], transmissible gastroenteritis coronavirus (TGEV) [28], and porcine epidemic diarrhea virus (PEDV) [29]. Differently from the involvement of p38 signaling pathway in inflammation, activation of the JNK pathway is involved in apoptosis and inflammation during coronavirus infection. JNK phosphorylation has been determined in cells infected with MHV [19, 30], SARS-CoV [19, 31–34], PEDV [29], and IBV [35].

The MAPK-ERK pathway comprises three core kinases-Raf, MAPK/ERK kinase (MEK), and ERK, which transmit extracellular signals into the intracellular environment to trigger cellular growth responses [36, 37]. After stimulation of cells by growth factors, chemokines, or serum, the GTP-binding protein Ras induces phosphorylation and activation of Raf, which in turn activates MAPK/ERK kinases 1 and 2 (MEK1/2), eventually activating ERK1/2 by phosphorylation. Activated ERK phosphorylates numerous substrates in different cellular compartments, leading to increased nucleotide synthesis, RNA transcription, and protein synthesis, enhanced cell cycle progression and proliferation, finally promoting cell survival [38]. Thus, ERK1/2 signaling axis controls various fundamental cellular events and affects the physiological environment of cells, thereby regulating the process of viral infection at certain stages of the viral life cycle, such as entry, viral gene transcription, protein expression or release of progeny virions [39–41]. More and more evidence suggest that virus infection activates MAPK pathway for efficient virus replication [42]. Thus, ERK1/2 is an attractive target for viruses to facilitate replication and survival. Differently from the p38 and JNK pathway, the interaction between coronavirus and ERK1/2 signaling has been less characterized. Xia et al. showed that activation of PI3K/Akt and ERK signaling pathways via TGF-β in IPEC-J2 cells is critical for the TGEV mediated epithelial-mesenchymal transition, and thus the secondary pathogen enterotoxigenic *Escherichia Coli* can more easily adhere to generating cells [43, 44]; SARS-CoV papain-like protease suppressed α interferon-induced responses through downregulation of ERK1 [45]; whereas accessory protein 3b induces AP-1 transcriptional activity and promotes inflammation through activation of JNK and ERK pathways [34]; Fung and Liu reported that ERK1/2 signaling was triggered by IBV infection and contributed to IBV-induced autophagy [46]; the ER stress induced expression of GADD153 promotes apoptosis by restricting the activation of ERK1/2 during IBV infection [47].

The dual-specific phosphatase family (DUSP), a subclass of protein tyrosine phosphatases, belongs to the mitogen-activated protein kinase (MAPK) phosphatase family and is primarily involved in the negative feedback regulation of MAPK-type pathway activity [48, 49]. DUSP constitute a structurally distinct family of 11 proteins, which perform their dephosphorylation activity on both phospho-threonine and phospho-tyrosine residues of the activated MAPK. Previous research has reported that IBV has developed a strategy to counteract the excessive induction of IL-6 and IL-8 in the infected Vero, H1299, and Huh7 cells, by inducing the expression of DUSP1, a negative regulator of the p38 MAPK [27]. Previous data have shown that the MEK/ERK axis exerts a retro-control on its own signaling through transcriptional and post-translational regulation of DUSP6 [50]. However, whether DUSP is involved in regulation of ERK1/2 signaling during coronavirus infection is largely unknown.

In this study, we find that IBV Beaudette strain infection activates ERK in different permissive cell lines, which may provide suitable intracellular environment for virus replication. Indeed, the expression of DUSP6 is upregulated during IBV infection, which is responsible for attenuation of ERK1/2 signaling, promotion of cell death, and limitation of virus replication. These data provide additional insights into the interaction of IBV with host cells, which may open up new avenues for coronavirus therapeutics.

## Materials and methods

### Cells and viruses

Vero and DF-1 cells were maintained in Dulbecco modified Eagle medium (DMEM) with 4500 mg/L glucose, supplemented with 10% fetal bovine serum (FBS) (Hyclone, USA) in the presence of 100 units/mL penicillin and 100 μg/mL streptomycin. (Invitrogen, USA). H1299 cells were maintained in RPMI 1640, supplemented with 10% FBS in the presence of penicillin and streptomycin. The above cells were purchased from ATCC (USA) and cultured at 37 °C with 5% CO_2_.

The Beaudette strain of IBV (ATCC VR-22) adapted to Vero cells was used in this study. Virus stock was prepared by infecting monolayers of Vero cells with multiplicity of infection (MOI) of 0.1. After attachment for 1 h (h), the unbound virus was removed and replaced with serum-free DMEM. The virus and cells were incubated at 37 °C and harvested when 100% cytopathic effect (CPE) were observed. After three freeze-thawing cycles, cell debris were removed by centrifugation at 5000 × *g* for 15 min, the supernatant was aliquoted and stored at − 80 °C as virus stock. A control of Vero cell lysates from mock-infected cells was prepared in the same manner.

### Inhibitors and antibodies

The MEK1//2 inhibitor U0126 (9903S) was purchased from Cell Signaling Technology (USA), DUSP6 inhibitor BCI (B4313) were purchased from Sigma-Aldrich (USA). Anti-IBV N antibody was produced through immunization of rabbit with N antigen. Anti-DUSP6 (#39441), anti-Flag (#14793), anti-phospho-ERK1/2 (#4370), anti-ERK1/2 (#4695), anti-poly (ADP-ribose) polymerase (PARP) (#5625), anti-β-actin (#3700), horseradish peroxidase (HRP)-conjugated anti-mouse or anti-rabbit IgG antibodies were purchased from Cell Signaling Technology (USA). Anti-PARP recognizes full length PARP (PARP-FL) and cleaved PARP (PARP-C).

### Pharmacological treatment

To test the effect of various pharmacological inhibitors on IBV infection, Vero, H1299, and DF-1 cells were seeded on 6-well plates at 5 × 10^5^ cells/well and cultured for 24 h until the cells reached 100% of confluence. The cells were infected with IBV at an MOI of 1 in serum-free medium and the inoculum was removed after 1 h, replaced with fresh medium containing 10 μM U0216 or 10 μM BCI. At 20 and 24 hpi, the expression of DUSP6 was determined with quantative RT-PCR, the activation of ERK1/2 and IBV N synthesis was monitored by Western blot.

### RNA isolation and northern blot analysis

Vero and H1299 cells were seeded in 100-mm-diameter dishes and infected with IBV at MOI of 1, respectively. Cells were harvested at 4 h intervals throughout the infection time course (0–20 h post-infection, hpi). Total RNA was isolated from the cells by use of Trizol reagent (Invitrogen) as recommended by the manufacturer. Briefly, cells were lysed in Trizol before a one-fifth volume of chloroform was added. The mixture was then incubated for 5 min at room temperature and centrifuged at 13 000 × rpm for 15 min at 4 °C. The aqueous phase was then mixed with equal volume of 100% isopropanol and incubated at –20 °C for 20 min. RNA was precipitated by centrifugation at 13,000 × rpm for 10 min at 4 °C. RNA pellets were washed with 70% RNase-free ethanol and dissolved in RNase-free ddH_2_O.

Northern blot probe was obtained by reverse transcription-PCT (RT-PCR) and labeled with digoxigenin (DIG) using a DIG labeling kit (Roche). Briefly, 2 μg of total RNA was used to perform reverse transcription using Expand reverse transcriptase (Roche). cDNA were then subjected to PCR using appropriate primers. Primers used for human DUSP6 were forward 5′-CCGTCACGGTGACAGTGGCTTA-3′ and reverse 5′-CTGCTGTGCGGGGACACGATT-3’.

To analyze RNA expression by northern blotting, 30 μg of RNA from each sample preparation was separated by electrophoresis on a 1.3% agarose formaldehyde gel and visualized using ethidium bromide staining and UV light. RNA was transferred onto a Hybond-N + membrane (Amersham Biosciences) and hybridized with DIG-labeled DUSP6 DNA probe overnight at 50 °C. After hybridization and stringent washes, the membrane was rinsed briefly (5 min) in washing buffer and blocked in blocking buffer for 30 min, after which the membrane was incubated with DIG antibody (Roche) for 30 min, washed twice for 15 min in washing buffer, and equilibrated for 3 min in detection buffer. The signal was detected with CDP-Star (Roche) according to the manufacturer's instructions.

### Quantitative real time RT-PCR

The level of DUSP6 mRNA was determined by quantitative real time RT-PCR. Briefly, 3 µg of total RNA was used to perform reverse transcription using expand reverse transcriptase (Roche, USA) and oligo-dT primer. Equal volume of cDNA was then PCR-amplified using SYBR green PCR master kit (Dongsheng Biotech, Guangdong, China). The DUSP6 and β-actin primers used in PCR were the following: DUSP6 forward primer 5′-GAAATGGCGATCAGCAAGACG-3′; DUSP6 reverse primer 5′-CGACGACTCGTATAGC TCCTG-3′; β-actin forward primer 5′-GATCTGGCACCACACCTTCT-3′; β-actin reverse primer 5′-GGGGTGTTGAAGGTCTCAAA-3′.

The relative copy number of DUSP6 mRNA were normalized to β-actin using the comparative cycle threshold values. Data were analyzed relative to the mock infection control group. All assays were performed in three replicates. All statistical analyses and calculations were performed using Graph Pad Prism 5 (Graph Pad Software Inc., La Jolla, CA, USA). The results are presented as means ± standard deviations (SD) as indicated. Student t test was used to compare data from pairs of treated or untreated groups. Statistical significance is indicated in the figure legends.

### Western blot analysis

Cells were harvested at the indicated infection time points and lysed with 2 × SDS loading buffer in the presence of 100 mM dithiothreitol and denatured at 100 °C for 5 min. Equivalent amounts of protein were separated by SDS-PAGE, followed by transferring onto polyvinylidenedifluoride (PVDF) membranes (Bio-Rad Laboratories, USA) by electroblotting. Immunoblot analysis was then performed by incubating membranes with blocking buffer (5% BSA in PBST) for 1 h at room temperature and incubating with appropriate antibodies diluted in blocking buffer for 1 h. After washing thrice with PBST, membranes were incubated with HRP-conjugated secondary antibody for 1 h and washed with PBST thrice. Blots were developed with an enhanced chemiluminescence (ECL) detection system (GE Healthcare Life Sciences, USA) and exposed to Chemiluminescence gel imaging system (Tanon 5200, Shanghai, China). The antibodies on the PVDF membranes were removed with stripping buffer (10 mM β-mercaptoethanol, 2% SDS, 62.5 mM Tris–Cl, pH 6.8) at 55 °C for 30 min before the membranes were re-probed with other antibodies.

### Plasmid construction

DUSP6 was amplified by PCR from HeLa cDNA and cloned into vector pCMV-HA between restriction enzymes *Hind* III and *Kpn* I under the control of a cytomegalovirus promoter, generating pCMV-HA-DUSP6 with HA-tag at the N-terminus. The dominant negative mutant pCMV-HA-DUSP6-DN (C293S) was generated using a Site Directed Mutagenesis Kit (Beyotime, Jiangsu, China). The primers for amplification of DUSP6 were 5′-AAGCCAACACCCTTCCAGTA-3′ (forward) and 5′-GCCCAGCTCTCTCTGACACA-3′ (reverse). The primers for mutation of DUSP6 were 5′-GTTCTGCTATGAGCTAGCTGGAGA GCCCTTGGTC-3′ (forward) and 5′-GACCAAGGGCTCTCCAGCTAGCTCATAGCAGAAC-3′ (reverse).

### Plasmid transfection and siRNA transfection

Cells grown in 6-well plates were transfected with 3 µg of pCMV-HA, pCMV-HA-DUSP6, or pCMV-HA-DUSP6-DN by lipofectamine 2000 according to the manufacturer’s instructions (Invitrogen). After 24 h post-transfection, cells were infected with IBV at an MOI of 1 and subjected to Western blot at 20 and 24 hpi.

To knock down DUSP6, cells were seeded onto 6-well plates. The small interfering RNA (siRNA) targeting to DUSP6 (siDUSP6) or non-targeting siRNA (sic) were transfected into the cells by using Lipofectamine 2000 according to the manufacturer’s instructions. At 36 h post-transfection, cells were infected with IBV at an MOI of 1. At 20 and 24 hpi, the knock down effect of DUSP6 was determined with quantative RT-PCR and the levels of corresponding proteins were measured by Western blot analysis. The siRNA sequences targeting to different sequences of DUSP6 were siDUSP6-1 5′-GGAGGGAAGUUACAUAUUATT-3′ and siDUSP6-2 5′-GGACAUCGA GUCUGACCUUTT-3′; non-targeting siRNA sequence was sic 5′-AUGUUCUAAUGCA CGCUGCTT-3′.

### TUNEL assay

The TUNEL assay was performed to label the 3′-end of fragmented DNA with fluorescein-dUTP in apoptotic cells. Vero, H1299, and DF-1 cells were grown on coverslips and infected with IBV Beaudette strain at an MOI of 1 in serum-free medium, the inoculum was removed after 1 h., replaced with fresh medium or medium containing 10 μM U0216, 10 μM BCI, or DMSO, and harvested at 24 hpi. The cells were washed with phosphate-buffered saline (PBS) and fixed with 4% paraformaldehyde for 15 min at room temperature. After washing with PBS, the cells were permeabilized with 0.5% Triton X-100 for 10 min and blocked with 3% FBS for 30 min at 37 °C. The TUNEL assay was carried out by Click-iTTM Plus TUNEL Apoptosis Assay Kit according to the manufacture’s instruction. The images of TUNEL positive cells were captured by a fluorescence microscope.

### One step growth curve and Tissue culture infectious dose 50 (TCID_50_) assay

Vero, H1299, and DF-1 cells were inoculated with IBV at an MOI of 5 for 1 h and replaced with fresh serum-free medium. The culture supernatants were harvested at 4, 8, 12, 16, 20, 24, and 28 hpi, respectively. The virus titers were determined by TCID50 as described previously. In brief, cells were seeded in 96-well plates at a density of 2.0 × 10^4^ cells per well. After 24 h, cells were infected with IBV, which was serially diluted tenfold using serum free medium. The virus and cells were incubated at 37 °C for 4 days. The cytopathic effect of cells was observed under microscopy. The TCID_50_ is calculated using Reed and Munch mathematical analysis [51].

### Statistical analysis

The statistical analysis was analyzed with Graphpad Prism8 software. The data are shown as means ± standard deviation (SD) of three independent experiments. Significance was determined with the Student test. *P* values < 0.05 were deemed statistically significant.

### Densitometry

The intensities of corresponding bands were quantified using the Image J program (NIH, USA) according to the manufacturer’s instruction.

## Results

### IBV infection activates the ERK signaling pathway

**Figure 1:**
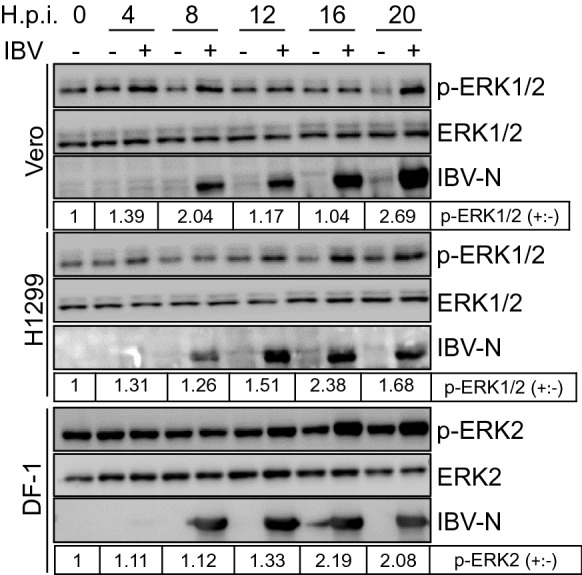
**Infection with IBV results in activation of ERK signaling in Vero, H1299 and DF-1 cells.** Vero, H1299, and DF-1 cells were infected with IBV-Beaudette at MOI of 1 or mock infection. At the indicated hours post-infection (hpi), cells were collected and lysed for Western blot analysis to detect phospho-ERK1/2 (p-ERK1/2), ERK1/2, and IBV N protein. The experiments were repeated in triplicate and the representative figures are shown. The signal of protein bands was determined by Image J. The intensities of p-ERK1/2 or p-ERK2 were normalized to total ERK1/2 or total ERK2. The ratio of p-ERK in IBV infected cells to mock infected cells are shown as p-ERK1/2 (+ :−) or p-ERK2 (+ :−).

Many DNA viruses are known to induce cellular signaling via the MAPK-ERK pathway, as they need to drive cells into a proliferative state to use the DNA synthesis machinery for their own replication. In contrast, the consequences of RNA virus-induced Raf/MEK/ERK signaling are less clear. Here, we analyzed whether IBV infection regulates the ERK signaling pathway and the biological consequences on virus replication. Vero, H1299 cells, chicken fibroblast DF-1 cells, which are permissive to the IBV Beaudette strain, were used in this study. Cells were either mock-infected or innoculated with IBV beaudette strain at an MOI of 1, and the cell lysates were collected at different time points post-infection. The phosphorylation level of ERK and total ERK was assessed by Western blot analysis. The successful replication of IBV was monitored by detection of viral N protein. As shown in Figure 1, although there was a basal level of phospho-ERK in all three cell types, IBV infection gradually increased the level of phospho-ERK along the infection time course and reached a peak at 16 and 20 h post-infection (hpi), compared to the mock-infected group. It was noted that the human anti-ERK1/2/ antibody only cross-reacted with the 42-kDa ERK2 isoform in DF-1 cells. There was no EKR1 detected, which was consistent with a previous report [52]. These results suggest that IBV infection activates the ERK signaling pathway, which is not restricted to a cell type.

### Pharmacological inhibition of ERK promotes apoptosis and impairs IBV replication

**Figure 2:**
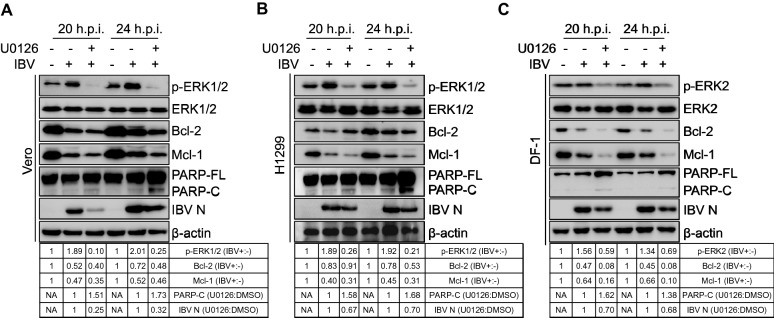
**Pharmacological suppression of MEK1/2 attenuates ERK1/2 signaling, promotes apoptosis, and impairs IBV replication.** Vero (**A**), H1299 (**B**), and DF-1 (**C**) cells were pre-incubated with U0126 (10 μM) or DMSO for 1 h, followed by IBV infection. 10 μM U0126 or DMSO was present throughout the infection. Mock infection was included as another control group. Cells were collected at 20 and 24 hpi and subjected to Western blot analysis. p-ERK1/2, ERK1/2, PARP, Bcl-2, Mcl-1, IBV N, and β-actin were detected. β-actin was included as a loading control. (PARP-FL: full-length PARP; PARP-C: cleaved PARP). The experiments were repeated in triplicate and the representative figures are shown. The signal of protein bands was determined by Image J software. The intensities of p-ERK1/2 or p-ERK2 were normalized to total ERK1/2 or total ERK2, the intensities of Bcl-2, Mcl-1, IBV N were normalized to β-actin, and the intensities of PARP-C were normalized to PARP-FL. The ratio of p-ERK1/2, p-ERK2, Bcl-2, and Mcl-1 of IBV infected cells to mock infected cells are shown as p-ERK1/2 (IBV + :−), p-ERK2 (IBV + :−), Bcl-2 (IBV + :−), and Mcl-1 (IBV + :−). The ratio of PARP-C and IBV N in U0126 treated cells to DMSO treated cells are shown as PARP-C (U0126:DMSO) and IBV N (U0126:DMSO).

To investigate the biological consequences of the ERK signaling pathway on IBV infection, we used U0126, an inhibitor that prevents the phosphorylation of ERK by specifically abrogating the activity of MEK1/2, to treat IBV-infected cells. Vero, H1299, and DF-1 cells were mock-infected or infected with IBV at an MOI of 1, followed by treatment with 10 μM U0126 or solvent DMSO (control). The levels of phospho-ERK and IBV N were detected by Western blot analysis at 20 and 24 hpi. As shown in Figure 2A–C, basal levels of phospho-ERK were visible in mock infected cells, IBV infection increased the level of phospho-ERK; however, U0126 treatment blocked the phosphorylation of ERK to undetectable levels; meanwhile, the synthesis of IBV N protein was reduced by the U0126 treatment, compared to the DMSO-treated group. These data show that inhibition of ERK by U0126 impairs IBV replication, suggesting that the activation of the ERK signaling pathway supports efficient IBV replication.

We next investigated whether activation of ERK was involved in protecting cells from IBV induced apoptosis. Apoptosis was monitored by assessing the cleavage of marker protein PARP and by measuring the fragmentation of cellular DNA with the TUNEL assay. As shown in Figure 2A–C, at 20 and 24 hpi, IBV infection triggered slight cleavage of PARP in Vero, H1299, and DF-1 cells. Additional file 1 shows that IBV infection triggered the fragmentation of cellular DNA at 24 hpi in all three cell types, which was labeled with Alexa Fluor 488 dye-dUTP. Thus, IBV infection indeed induced apoptosis in different cell lines. It was noted that although U0126 blocked the phosphorylation of ERK in Vero, H1299, and DF-1 cells, it did not trigger PARP cleavage in the absence of virus infection (Additional file 2). TUNEL assay in Additional file 1 also shows that U0126 did not directly trigger DNA fragmentation. Thus, treatment with U0126 did not trigger apoptosis directly in all three cell types used in this study. However, in the presence of IBV infection, U0126 treatment promoted both cleavage of PARP (Figure 2) and DNA frangmentation (Additional file 1), suggesting that pharmacological inhibition of ERK promotes apoptosis during virus infection. We further examined the levels of pro-survival Bcl2 family protein Bcl-2 and Mcl-1. The results show that IBV infection decreased the levels of both Bcl-2 and Mcl-1, and U0126 treatment further reduced the levels of these two pro-survival proteins (Figure 2). It is worth noting that U0126 treatment did not directly reduce the levels of Bcl-2 and Mcl-1 in the absence of IBV infection, compared to the DMSO-treated group (Additional file 2). Thus, inhibition of ERK promotes IBV-induced apoptosis, probably via regulation of Bcl-2 and Mcl-1 levels. The activation of ERK probably facilitates efficient IBV replication by prolonging cell survival.

### IBV infection induces the expression of DUSP6

**Figure 3:**
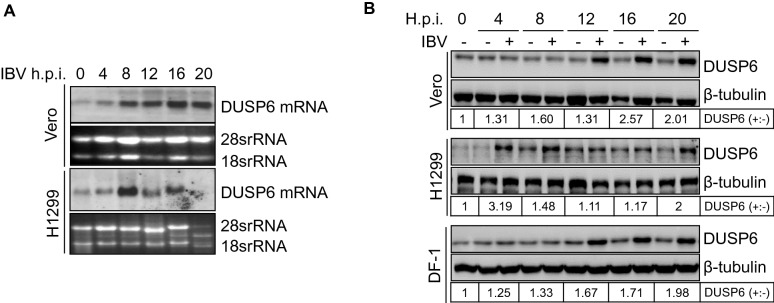
**Up-regulation of DUSP6 by IBV infection.** **A** Northern blotting analysis of DUSP6 in IBV-infected Vero and H1299 cells. Vero and H1299 cells were infected with IBV at MOI of 1 and harvested at the indicated time points. Total RNA were extracted and were separated on agarose gel, followed with northern blotting analysis by using specific DIG-labeled DUSP6 probe. **B** Vero, H1299 and DF-1 cells were infected with IBV or mock infection and harvested at the indicated time points. Cell lysates were subjected to Western blot analysis by using anti-DUSP6 antibody. β-tubulin was detected as the loading control. The experiments were repeated in triplicate and the representative figures are shown. The signal of protein bands was determined by Image J software. The intensities of DUSP6 were normalized to total β-tubulin. The ratio of DUSP6 in IBV infected cells to mock infected cells are shown as DUSP6 (+ :−).

DUSP6 negatively regulates MAPK signaling by dephosphorylating tyrosine or serine/threonine residues on phospho-MAPK. To examine whether IBV infection induces the expression of DUSP6, Vero and H1299 cells were infected with IBV at an MOI of 1 and subjected to northern blot analysis by using DIG-labeled DUSP6 probe. As shown in Figure 3A, in Vero cells, DUSP6 mRNA gradually increased over infection time from 4 to 20 hpi; in H1299 cells, DUSP6 mRNA was induced at 4 hpi, peaked at 8 hpi, and decreased over infection time from 8 to 20 hpi. It was worth noting that DUSP6 mRNA was invisible at 20 hpi in H1299 cells; meanwhile, total RNA was degraded at this time point, as evidenced with the reduced signals of 28 s rRNA and 18 s rRNA and presence of additional bands. This can be attributed to fast replication of IBV and early cell death in H1299 cells (Additional file 3). Western blot confirmed the upregulation of DUSP6 at protein level in IBV-infected Vero, H1299, and DF-1 cells (Figure 3B). The induction kinetics of DUSP6 was slightly different among the three cell types: in Vero and DF-1 cells, DUSP6 protein was gradually induced over infection time from 12 to 20 hpi; however, in H1299 cells, the induction of DUSP6 was much earlier than the two other cell lines, as early as 4 hpi, which was due to the faster replication of virus in H1299 cells than the other two cell lines (Additional file 3). It has been reported that H1299 cells were more susceptible to IBV infection than the other cells [53]. These data demonstrate that IBV infection promotes the expression of DUSP6 at both mRNA and protein levels.

### DUSP6 negatively regulates ERK signaling, promotes apoptosis, and suppresses IBV replication

**Figure 4:**
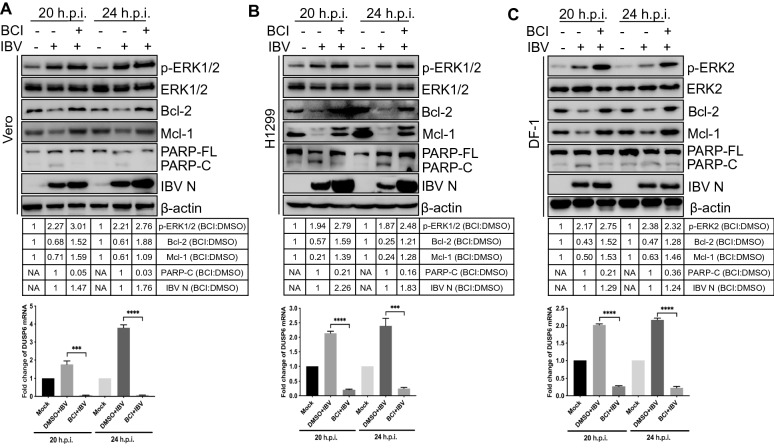
**Pharmacological inhibition of DUSP6 promotes ERK1/2 signaling, reduces apoptosis, and facilitates IBV replication.** Vero (**A**), H1299 (**B**), and DF-1 (**C**) cells were pre-incubated with BCI (10 μM) or DMSO for 1 h, followed by IBV infection. 10 μM BCI or DMSO was present throughout the experiment. Mock infection was included as another control group. Cells were harvested at 20 and 24 hpi and subjected to cell lysis or RNA extraction. The level of p-ERK1/2, ERK1/2, PARP, Bcl-2, Mcl-1, IBV N, and β-actin were checked by Western blot analysis (upper panels). The expression of DUSP6 was analyzed by quantitative real time RT-PCR (low panels). The experiments were repeated in triplicate and the representative data are shown. The signal of protein bands was determined by Image J software. The intensities of p-ERK1/2 or p-ERK2 were normalized to total ERK1/2 or total ERK2, the intensities of Bcl-2, Mcl-1, IBV N were normalized to β-actin, and the intensities of PARP-C were normalized to PARP-FL. The ratio of PARP-C and IBV N in BCI treated cells to DMSO treated cells are shown as PARP-C (BCI:DMSO) and IBV N (BCI:DMSO). The bar graphs in the low panels show means ± SD of three independent determination of relative expression of DUSP6 mRNA. *P* values were calculated by Student test. ***P < 0.001, ****P < 0.0001 (highly significant).

We next examined the effect of DUSP6 on ERK signaling. Vero, H1299, and DF-1 cells were infected with IBV at MOI of 1 and treated with 10 μM BCI, an inhibitor of DUSP6, and subjected to Western blot analysis to examine ERK signaling at 20 and 24 hpi. As shown in Figure 4A–C (upper panels), in all three cell types, blockage of DUSP6 activity by BCI increased the level of phospho-ERK, compared to those cells receiving IBV only, suggesting that DUSP6 might be involved in dephosphorylating ERK. In all three cell typres, the augmentation of ERK signaling by BCI resulted in the decrease of PARP cleavage (Figure 4) and produced less TUNEL positive cells during IBV infection (Additional file 1), accompanied by recovery of Bcl-2 and Mcl-1 levels, although IBV replication was promoted, as evidenced by the increased synthesis of IBV N protein. In the absence of IBV infection, BCI treatment neither changed the level of Bcl-2/Mcl-1 nor triggered PARP cleavage (Additional file 2)/DNA fragmentation (Additional file 1). It was noted that BCI treatment reduced the IBV-induced transcription of DUSP6, as determined by quantitative real time RT-PCR (Figure 4A–C, low panels), although the underlying mechanism is unclear. These data demonstrate that blockage of DUSP6 activity protects cells from death by augmenting ERK signaling and supports virus replication.

**Figure 5:**
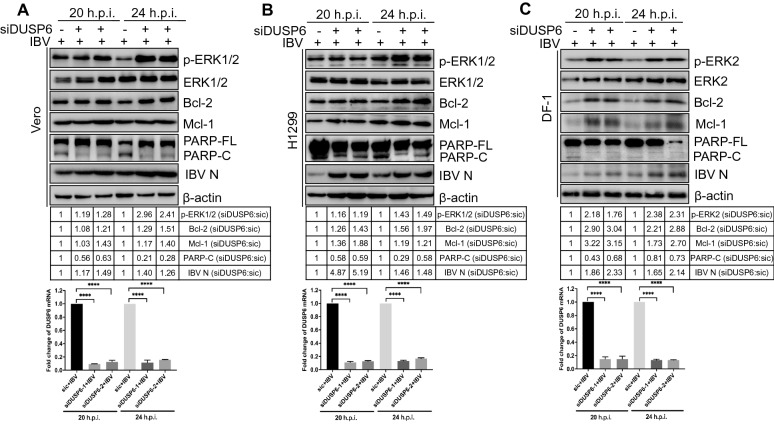
**Specific knock down of DUSP6 increases the phosphorylation of ERK1/2, reduces apoptosis, and promotes IBV replication.** Vero (**A**), H1299 (**B**), DF-1 (**C**) cells were transfected with non-target siRNA (sic), siDUSP6-1 or siDUSP6-2 for 36 h, followed with IBV infection. Cells were harvested at 20 and 24 hpi and subjected to cell lysis or RNA extraction. The knock down effect of DUSP6 was analyzed by quantitative real time RT-PCR (low panel). The level of p-ERK1/2, ERK1/2, PARP, Bcl-2, Mcl-1, IBV N, and β-actin were checked with Western blot analysis. The experiments were repeated in triplicate and the representative data are shown. The signal of protein bands was determined by Image J software. The intensities of p-ERK1/2 or p-ERK2 were normalized to total ERK1/2 or total ERK2, the intensities of Bcl-2, Mcl-1, IBV N were normalized to β-actin, and the intensities of PARP-C were normalized to PARP-FL. The ratio of p-ERK1/2, p-ERK2, Bcl-2, Mcl-1, PARP-C, IBV N in siDUSP6 transfected cells to sic transfected cells were shown as (siDUSP6:sic). The bar graphs in the low panels show means ± SD of three independent determinations of relative expression of DUSP6 mRNA. *P* values were calculated by Student test. ****P < 0.0001 (highly significant).

To confirm the above conclusion, we specifically depleted DUSP6 by siRNA. Vero, H1299, and DF-1 cells were transfected with non-targeting siRNA (sic) and two strands of siDUSP6 targeting different sequences of DUSP6 (siDUSP6-1, siDUSP6-2), respectively, were infected with IBV at MOI of 1 for 20 or 24 h. The knock down efficiency of DUSP6 was measured by quantitative real time RT-PCR. As shown in Figure 5A–C (low panels), successful silence of DUSP6 was obtained in all three cell types. Depletion of DUSP6 increased the level of phospho-ERK, especially at 24 hpi; the cleavage of PARP was attenuated, the levels of Bcl-2 and Mcl-1 were recovered, IBV N protein synthesis was increased (Figure 5A–C, upper panels). These data confirm that interfered expression of DUSP6 augments IBV triggered ERK signaling, protects cells from death, and supports efficient IBV replication. The induction of DUSP6 by IBV infection negatively regulates ERK signaling and promotes cell death, thereby playing an anti-viral role.

**Figure 6:**
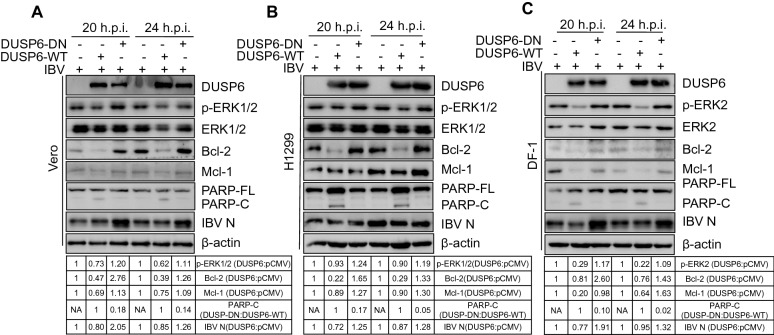
**The phosphatase activity of DUSP6 is sufficient for suppressing ERK1/2 signaling, promoting apoptosis, and impairing IBV replication.** Vero (**A**), H1299 (**B**), and DF-1 (**C**) cells were transfected with constructs encoding HA tagged DUSP6-WT and DUSP6-DN, or vector pCMV-HA, respectively. At 24 h post-transfection, cells were infected with IBV (MOI = 1) for 20 and 24 h. The expression of HA-DUSP6-WT and HA-DUSP6-DN, the levels of p-ERK1/2, ERK1/2, PARP, Bcl-2, Mcl-1, IBV N, and β-actin were checked by Western blot analysis. The experiments were repeated in triplicate and the representative data were shown. The signal of protein bands was determined by Image J software. The intensities of p-ERK1/2 or p-ERK2 were normalized to total ERK1/2 or total ERK2, the intensities of Bcl-2, Mcl-1, IBV N were normalized to β-actin, and the intensities of PARP-C were normalized to PARP-FL. The ratio of p-ERK1/2, p-ERK2, Bcl-2, Mcl-1, and IBV N in DUSP6 or DUSP6-DN transfected cells to vector transfected cells are shown as (DUSP6:pCMV). The ratio of PARP-C in DUSP6 transfected cells to DUSP6-DN transfected cells are shown as PARP-C (DUSP6:DUSP6-DN).

**Figure 7:**
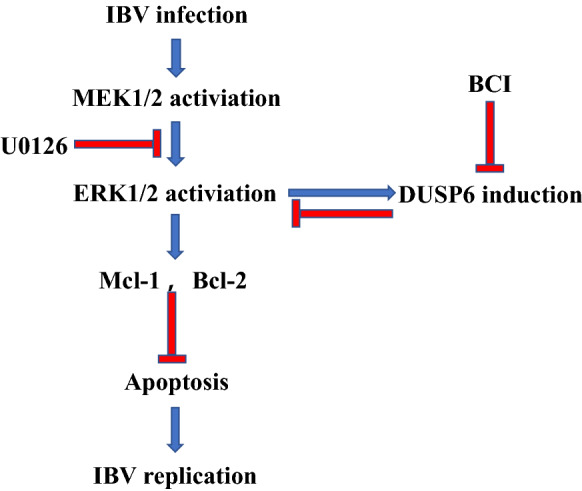
**The proposed working model.** IBV infection activates MEK1/2-ERK1/2 signaling, which protects cells from death by reducing the level of Mcl-1 and Bcl-2, thereby supporting efficient IBV replication. The activation of ERK1/2 pathway induces the phosphatase DUSP6 expression. DUSP6 forms a negative regulation loop by dephosphorylating ERK1/2, to restrict this prosurvival signal. The upregulation of DUSP6 promotes cell death and impairs IBV replication, representing one of the host antiviral responses. U0126 inhibits the activation of ERK1/2 and promotes cell death, thereby suppressing IBV replication, while BCI inhibits DUSP6 and promotes ERK1/2 signaling/cell survival, thereby promoting IBV replication.

The active site of DUSP6 is HCXXXXR, cysteine 293 plays an important role in the nucleophilic attack of phosphorus on ERK, whereas arginine 298 interacts directly with the phosphate group on phosphotyrosine or phosphothreonine for transition-state stabilization [54, 55]. DUSP6 dominant negative mutant C293S not only sequesters ERK away from endogenous DUSP6 but also restricts ERK to the cytoplasm via its selective, high-affinity interaction with ERK [56]. To further validate the role of DUSP6 on ERK signaling and IBV replication, we constructed wild type DUSP6 and dominant negative mutant DUSP6-DN (C293S), with HA tag at N-terminus. Vero, H1299, and DF-1 cells were transfected with PCMV-HA, PCMV-HA-DUSP6, and PCMV-HA-DUSP6-DN, respectively, followed by IBV infection. Cells were harvested at 20 and 24 hpi and subjected to Western blot analysis and TUNEL assay. As shown in Figure 6A–C, HA-DUSP6 and HA-DUSP6-DN were successfully expressed. Compared with the vector transfected group, overexpression of HA-DUSP6 greatly attenuated the phosphorylation of ERK; however, overexpression of HA-DUSP6-DN did not attenuate the level of phosphorylation of ERK, suggesting that this mutant loses phosphatase activity. In accordance, during IBV infection, HA-DUSP6 promoted greatly reduced Bcl-2 and Mcl-1 levels, and promoted the cleavage of PARP, whereas HA-DUSP6-DN had no obvious effect on the cleavage of PARP and the levels of Bcl-2 and Mcl-1. Moreover, HA-DUSP6-DN transfected cells produced less TUNEL positive cells during IBV infection, compared to the vector transfection group, probably due to the competitive interference of the pro-apoptotic function of endogenous DUSP6. Thus, the phosphatase activity of DUSP6 is indeed involved in attenuating the ERK signaling pathway. It is noteworthy that overexpression of HA-DUSP6 slightly impaired IBV replication; interestingly, compared to the vector-transfected group, HA-DUSP6-DN significantly increased IBV replication, probably by competitively interfering with the anti-viral function of the endogenous DUSP6. Altogether, DUSP6 phosphatase activity plays an anti-viral role by suppressing ERK signaling cascades and promotes cell death (Figure 7).

## Disscusion

Differential utilization of ERK signaling pathway by viruses highlights the importance of this pathway in regulating a wide variety of cellular fates that ultimately influence viral infection. The present study shows that IBV infection activates the ERK signaling pathway in various cell types, and this activation is required for protecting cells from death and the achievement of productive virus infection; meanwhile, the induction of DUSP6 in turn restricts the activation of the ERK signaling cascade, promotes cell death and impairs virus infection. The enzymatic active site of DUSP6 is sufficient to dephosphorylate ERK and to attenuate the signaling cascade.

Viruses usually alter ERK signaling in order to induce a proliferative state in the cell or prevent induction of cell death. This important signaling cascade is differently employed by various RNA viruses. Activation of ERK in virus-infected cells is a common phenomenon and usually favors the virus to enhance its own infection. For example, ERK1/2 is characterized as a virus-associated kinase to regulate human immunodeficiency virus (HIV) infectivity [57]; pseudorabies virus (PRV) glycoprotein E activates the ERK1/2 signaling pathway in T cells, resulting in T cell aggregation and migration [58]; the vaccinia virus O1 protein is required for sustained activation of ERK1/2 and promotes viral virulence [59, 60]; MEK1-ERK signal cascade is required for the efficient replication of Enterovirus 71 (EV71) [59, 61]; ERK1/2 signaling activation is involved in an efficient arenavirus RNA synthesis [62]; Newcastle disease virus (NDV) V Protein promotes viral replication in HeLa cells through the activation of MEK/ERK signaling [63]. In contrast, several studies have shown that certain viruses interfere with the ERK1/2 pathway to support an infectious or persistent infection. For example, herpes simplex virus (HSV) inhibits the activity of ERK1/2 by Us3 serine/threonine protein kinase [64]; dengue virus type 2 inhibits the activity of ERK1/2 to downregulate cytokine production [65]. Therefore, to facilitate their own infection, viruses have adopted different strategies to regulate the ERK signaling pathway. In this study, we observe that the activation of ERK signaling is required for prolonging cell survival and efficient IBV infection.

DUSP are a subset of protein tyrosine phosphatases, many of which dephosphorylate threonine and tyrosine residues on MAPK [66–68]. The regulated expression and activity of DUSP family members controls MAPK intensity and duration to determine the type of physiological response. In a previous study, we found that DUSP1 is up-regulated during IBV infection, negatively regulates the p38 signaling pathway and restricts the production of inflammatory factors [27]. In this study, another phosphatase of this family, DUSP6, which is responsible for dephosphorylating p-ERK1/2 [48, 69], was found to be upregulated during IBV infection. Inhibition of DUSP6 by chemical BCI or by siRNA enhances ERK1/2 signaling, protects cells from death, and promotes efficient IBV replication; overexpression of DUSP6 attenuates ERK1/2 signaling, promotes apoptosis, and impairs IBV infection; whereas expression of functional null DUSP6-DN has no effect on the ERK1/2 signaling and apoptosis, but facilitates efficient IBV replication. Thus, induction of DUSP6 probably is one of the anti-viral response strategies of host cells, which is involved in restricting ERK signaling and promoting cell death. In our previous study, we observed that ER stress induced GADD153 is also involved in restricting the ERK1/2 signaling and promoting apoptosis during IBV infection [47], suggesting cells employ multiple mechanisms to restricts ERK1/2 and limit virus infection. Whether DUSP6 is involved in immune response or amalgamator during IBV infection needs to be further investigated.

A recent kinome analysis suggests that ERK/MAPK and PI3K/Akt/ mTOR signaling pathways were specifically modulated by MERS-CoV infection [70]. Subsequent analysis demonstrates that kinase inhibitors targeting the ERK1/2 signal pathway (selumetinib and trametinib) inhibits MERS-CoV infection by ≥ 95% when added pre- or post-infection [70]. From a drug discovery perspective, MAPK are promising drug targets for manipulating MAPK-dependent responses, to either boost or subdue immune responses and cell death in infectious diseases. Here, we show the activation of ERK1/2 supports efficient replication of IBV, U0126 treatment blocks the ERK1/2 signaling and significantly impairs IBV replication. Thus, ERK signaling pathway is a potential target for therapeutic development. In summary, we find that activation of ERK1/2 signaling by IBV infection promotes cell survival and facilitates virus replication; in turn, the induction of DUSP6 is responsible for dephosphorylation of the activated ERK1/2 and shuts down the growth-stimulating signals, exerts anti-viral effect. The specific mechanism by which virus infection promotes ERK1/2 activation is unclear. Future research will show whether IBV proteins may be involved in ERK1/2 activation. Our findings add new knowledge to the regulatory mechanisms governing coronavirus-induced MAPK, highlighting a novel concept of anti-coronavirus therapy.

## Supplementary information

**Additional file 1.** Detection of IBV-induced apoptosis by TUNEL assay. Vero, H1299, and DF-1 cells were mock-infected for 24 h, or treated with 10 μM U0216 or 10 μM BCI for 24 h, or incubated with IBV Beaudette strain (MOI = 1) for 1 h and then treated with 10 μM U0216 or 10 μM BCI for 24 h, or transfected with DUSP6 or DUSP6-DN for 24 h and then infected with BV Beaudette strain (MOI = 1) for 24 h. Cells were subjected to TUNEL assay. The images of TUNEL positives cells were obtained by a fluorescence microscope.

**Additional file 2.** Treatment with U0126 or BCI alone does not trigger apoptosis directly. Vero, H1299, and DF-1 cells were incubated with DMSO, U0126 (10 μM), or BCI (10 μM) or 24 h. Cells were collected and subjected to Western blot analysis. p-ERK1/2, ERK1/2, PARP, Bcl-2, Mcl-1, IBV N, and β-actin were detected. β-actin was included as loading control. The intensities of p-ERK1/2 or p-ERK2 were normalized to total ERK1/2 or total ERK2, the intensities of Bcl-2, Mcl-1, IBV N were normalized to β-actin, and the intensities of PARP-C were normalized to PARP-FL. The ratio of p-ERK1/2, p-ERK2, Bcl-2, Mcl-1 of IBV infected cells to mock infected cells were shown as p-ERK1/2 (+:-), p-ERK2 (+:-), Bcl-2 (+:-), Mcl-1 (+:-). The ratio of PARP-C and IBV N in U0126 treated cells to DMSO treated cells were shown as PARP-C (+:-) and IBV N IBV N (+:-).

**Additional file 3.** Growth curve of IBV in Vero, H1299, and DF-1 cells. Cells were inoculated with IBV at MOI of 5 for 1 h and replaced with fresh serum-free medium. The culture supernatants were harvested at indicated times and titered by TCID_50_ in corresponding cell types. Error bars represent the standard deviation.

## Data Availability

All data generated or analysed during this study are included in this published article.

## Abbreviations

AP-1: activator protein-1
CPE: cytopathic effect
DN: dominant negative
DUSP6: dual-specific phosphatase 6
ERK: extracellular signal-regulated kinase
EV71: enterovirus 71
FCoV: feline coronavirus
HIV: human immunodeficiency virus
HSV: herpes simplex virus
IBV: infectious bronchitis virus
JNK: C-Jun N-terminal kinase
MAPK: mitogen-activated protein kinase
MEK: MAPK/ERK kinase
MERS-CoV: middle east respiratory syndrome coronavirus
MHV: mouse hepatitis virus
MOI: multiple of infection
NDV: Newcastle disease virus
PARP: poly ADP-ribose polymerase
PEDV: porcine epidemic diarrhea virus
PI3K: phosphatidylinositol 3-kinase
PRV: pseudorabies virus
RTC: replication/transcription complex
SARS-CoV: severe acute respiratory syndrome coronavirus
TGEV: transmissible gastroenteritis coronavirus
TGF-β: transforming growth factor-β
