# Supplementary material for: Upregulation of DUSP6 impairs infectious bronchitis virus replication by negatively regulating ERK pathway and promoting apoptosis
Source: Vet Res. 2021 Jan 11;52:7. doi: 10.1186/s13567-020-00866-x (PMC7798014; doi:10.1186/s13567-020-00866-x)
Supplement: Supplementary file 1 — Additional file 1. Detection of IBV-induced apoptosis by TUNEL assay. Vero, H1299, and DF-1 cells were mock-infected for 24 h, or treated with 10 μM U0216 or 10 μM BCI for 24 h, or incubated with IBV Beaudette strain (MOI = 1) for 1 h and then treated with 10 μM U0216 or 10 μM BCI for 24 h, or transfected with DUSP6 or DUSP6-DN for 24 h and then infected with BV Beaudette strain (MOI = 1) for 24 h. Cells were subjected to TUNEL assay. The images of TUNEL positives cells were obtained by a fluorescence microscope. [file 13567_2020_866_MOESM1_ESM.docx]

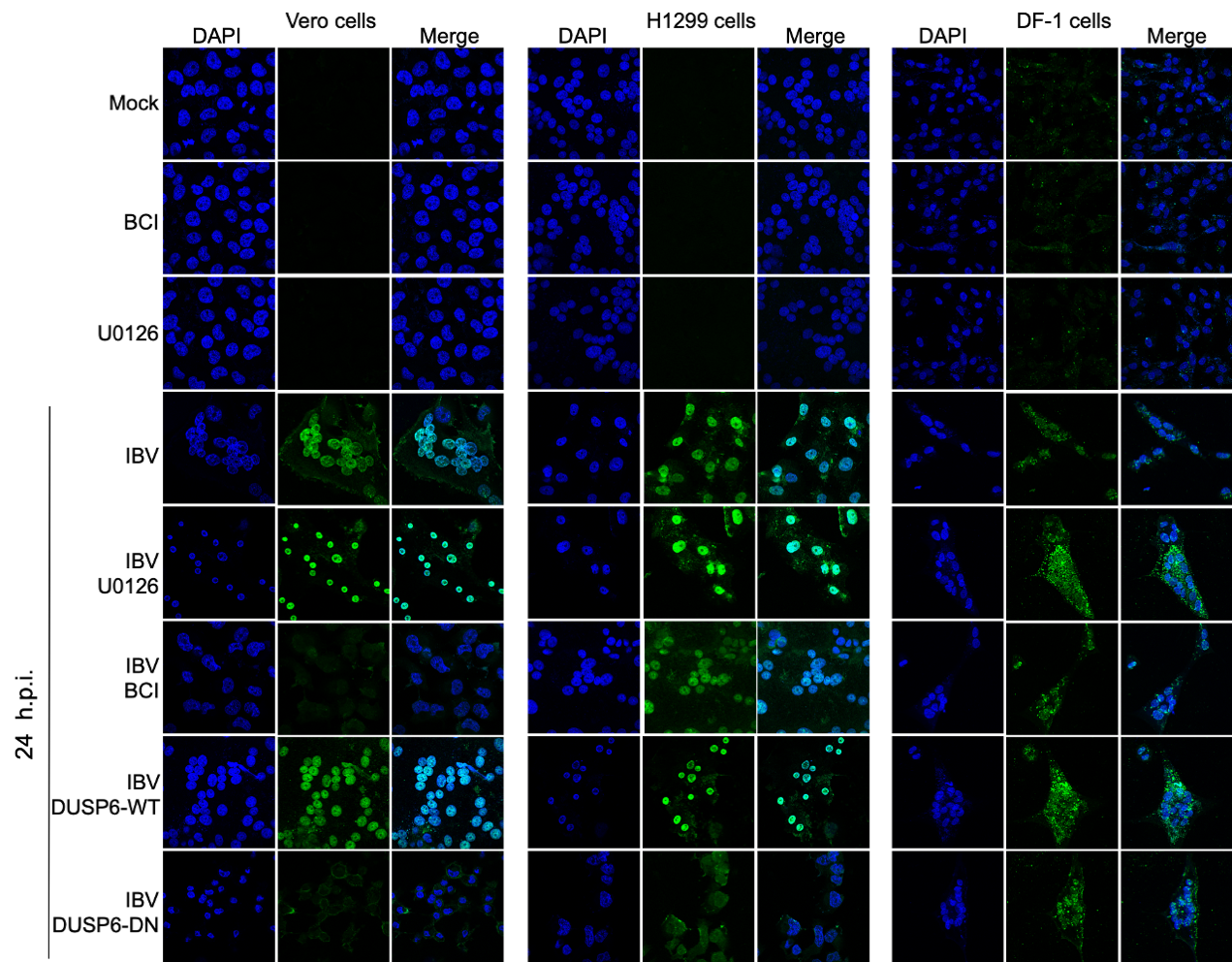


**Additional file 1.** Detection of IBV-induced apoptosis by TUNEL assay. Vero, H1299, and DF-1 cells were mock-infected for 24 h, or treated with 10 μM U0216 or 10 μM BCI for 24 h, or incubated with IBV Beaudette strain (MOI=1) for 1 h and then treated with 10 μM U0216 or 10 μM BCI for 24 h, or transfected with DUSP6 or DUSP6-DN for 24 h and then infected with BV Beaudette strain (MOI=1) for 24 h. Cells were subjected to TUNEL assay. The images of TUNEL positives cells were obtained by a fluorescence microscope.
