# Supplementary material for: Upregulation of DUSP6 impairs infectious bronchitis virus replication by negatively regulating ERK pathway and promoting apoptosis
Source: Vet Res. 2021 Jan 11;52:7. doi: 10.1186/s13567-020-00866-x (PMC7798014; doi:10.1186/s13567-020-00866-x)
Supplement: Supplementary file 2 — Additional file 2. Treatment with U0126 or BCI alone does not trigger apoptosis directly. Vero, H1299, and DF-1 cells were incubated with DMSO, U0126 (10 μM), or BCI (10 μM) or 24 h. Cells were collected and subjected to Western blot analysis. p-ERK1/2, ERK1/2, PARP, Bcl-2, Mcl-1, IBV N, and β-actin were detected. β-actin was included as loading control. The intensities of p-ERK1/2 or p-ERK2 were normalized to total ERK1/2 or total ERK2, the intensities of Bcl-2, Mcl-1, IBV N were normalized to β-actin, and the intensities of PARP-C were normalized to PARP-FL. The ratio of p-ERK1/2, p-ERK2, Bcl-2, Mcl-1 of IBV infected cells to mock infected cells were shown as p-ERK1/2 (+:-), p-ERK2 (+:-), Bcl-2 (+:-), Mcl-1 (+:-). The ratio of PARP-C and IBV N in U0126 treated cells to DMSO treated cells were shown as PARP-C (+:-) and IBV N IBV N (+:-). [file 13567_2020_866_MOESM2_ESM.docx]

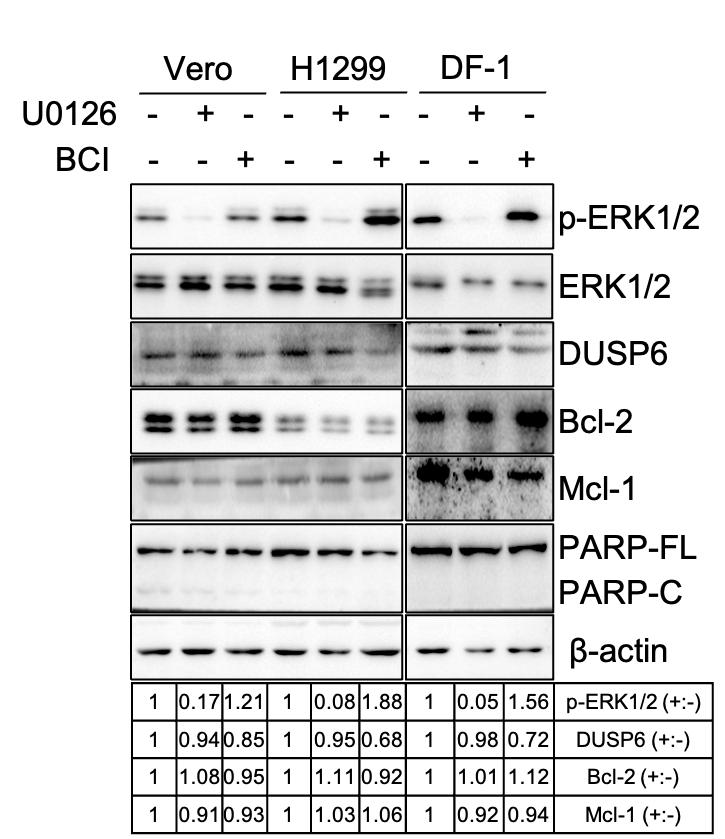


**Additional file 2**. Treatment with U0126 or BCI alone does not trigger apoptosis directly. Vero, H1299, and DF-1 cells were incubated with DMSO, U0126 (10 μM), or BCI (10 μM) or 24 h. Cells were collected and subjected to western blot analysis. p-ERK1/2, ERK1/2, PARP, Bcl-2, Mcl-1, IBV N, and β-actin were detected. β-actin was included as loading control. The intensities of p-ERK1/2 or p-ERK2 were normalized to total ERK1/2 or total ERK2, the intensities of Bcl-2, Mcl-1, IBV N were normalized to β-actin, and the intensities of PARP-C were normalized to PARP-FL. The ratio of p-ERK1/2, p-ERK2, Bcl-2, Mcl-1 of IBV infected cells to mock infected cells were shown as p-ERK1/2 (+:-), p-ERK2 (+:-), Bcl-2 (+:-), Mcl-1 (+:-). The ratio of PARP-C and IBV N in U0126 treated cells to DMSO treated cells were shown as PARP-C (+:-)and IBV N IBV N (+:-).
