# Supplementary material for: Upregulation of DUSP6 impairs infectious bronchitis virus replication by negatively regulating ERK pathway and promoting apoptosis
Source: Vet Res. 2021 Jan 11;52:7. doi: 10.1186/s13567-020-00866-x (PMC7798014; doi:10.1186/s13567-020-00866-x)
Supplement: Supplementary file 3 — Additional file 3. Growth curve of IBV in Vero, H1299, and DF-1 cells. Cells were inoculated with IBV at MOI of 5 for 1 h and replaced with fresh serum-free medium. The culture supernatants were harvested at indicated times and titered by TCID50 in corresponding cell types. Error bars represent the standard deviation. [file 13567_2020_866_MOESM3_ESM.docx]

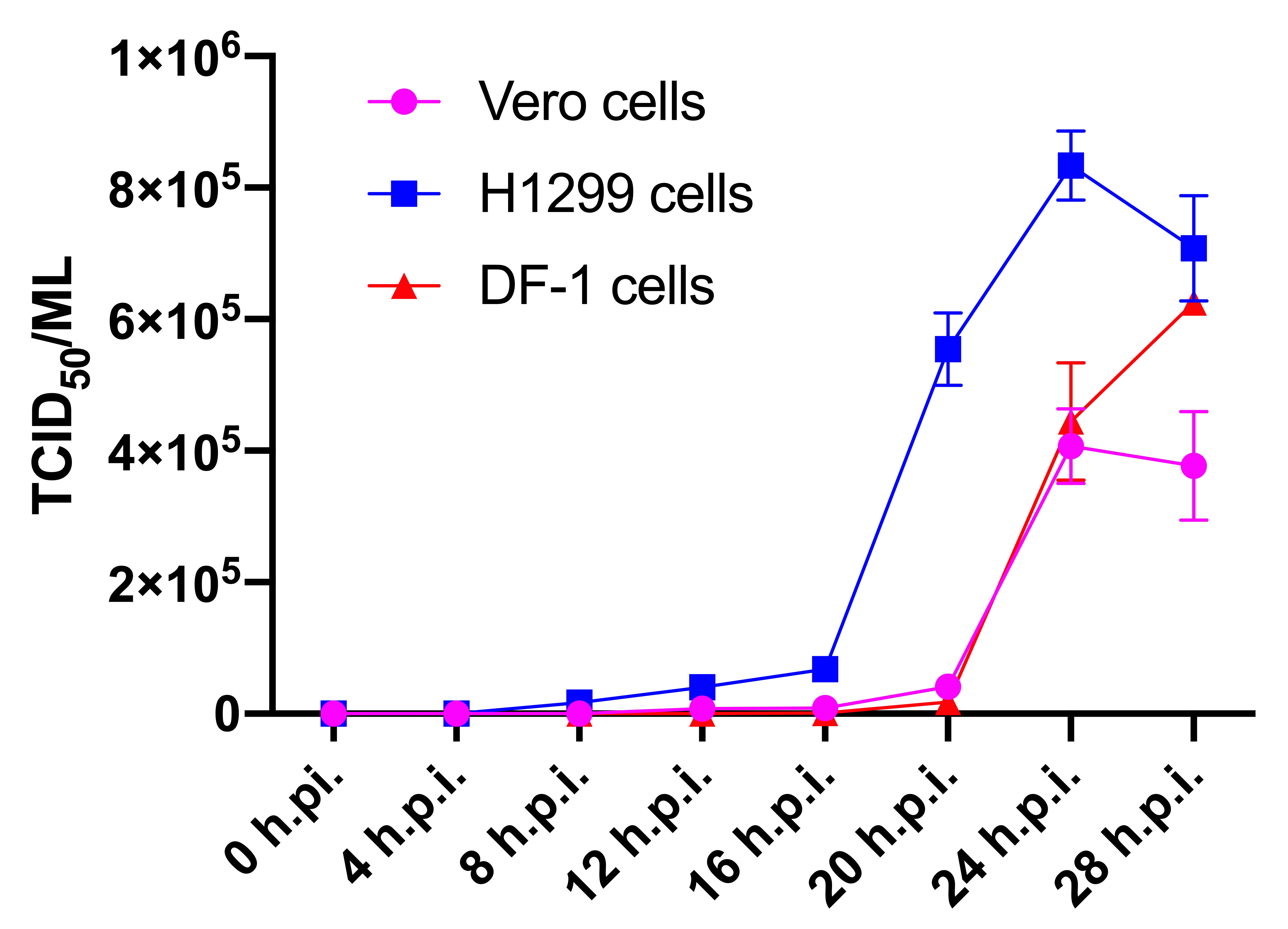


**Additional file 3.** Growth curve of IBV in Vero, H1299, and DF-1 cells. Cells were inoculated with IBV at MOI of 5 for 1 h and replaced with fresh serum-free medium. The culture supernatants were harvested at indicated times and titered by TCID_50_ in corresponding cell types. Error bars represent the standard deviation.
